# Assessment of the Daily Living Activities of Older People (2004–2023): A Bibliometric and Visual Analysis

**DOI:** 10.3390/healthcare12121180

**Published:** 2024-06-11

**Authors:** Ying Cui, Mankyu Choi

**Affiliations:** 1 Department of Public Health Science, Graduate School and Transdisciplinary Major in Learning Health Systems, Graduate School, Korea University, 145, Anam-ro, Seongbuk-gu, Seoul 02841, Republic of Korea; cuiyingg@korea.ac.kr; 2 School of Health Policy & Management, College of Public Health Science and Transdisciplinary Major in Learning Health Systems, Graduate School, Korea University, 145, Anam-ro, Seongbuk-gu, Seoul 02841, Republic of Korea

**Keywords:** activities of daily living, bibliometric analysis, older people, risk, trends

## Abstract

With a rapidly aging global population, comprehending the risks associated with older people’s activities of daily living is increasingly important; yet, interdisciplinary analyses remain rare. By providing a bibliometric overview of the capability risks associated with older people’s activities of daily living, in order to identify prevailing trends and future directions in the field, the study aims to fill this gap. Using CiteSpace software to analyze data from 928 articles published between 2004 and 2023, the study results demonstrate the growing interest in the capability risks of older people’s activities of daily living, with the United States leading in the number of publications, and geriatrics emerging as the dominant discipline. Notably, Institut National de la Sante et de la Recherche Medicale (Inserm) in France emerges as a pivotal contributor in the field. Key research topics encompass risk factors associated with a decline in daily activities and disease-related studies, with emerging trends in cognitive function and instrumental activity research. Future research should prioritize the development of predictive mechanisms for daily living trends, exploration of caregiving solutions, and promotion of interdisciplinary collaboration. This study highlights promising avenues for further research, emphasizing the importance of predictive modeling, innovative caregiving strategies, and interdisciplinary cooperation in addressing capability risks in the activities of daily living of older people.

## 1. Introduction

The World Health Organization predicts that by 2050, the global population aged 60 years and above will surpass two billion, signaling a major demographic transition toward societies characterized by aging populations [1]. With age progression, increasing challenges to independence arise owing to declines in physical function and alterations in social dynamics which affect activities of daily living (ADL) [2]. Surveys have highlighted that, globally, approximately 40% of older individuals face limitations or difficulties in maintaining their daily living capabilities [3]. Concurrent research underscores the association between ADL and physical decline [4], and mental health disorders [5]. Deterioration in the ability to engage in ADL increases the risk of falls [6,7], fractures [8], depression [9], and cognitive decline [10,11], all of which may elevate the likelihood of death. Falls, in particular, have become the second leading cause of accidental death worldwide [12]. Conversely, advanced age and age-related illnesses exacerbate the decline in these abilities, rendering older individuals increasingly dependent on assistance in undertaking their daily activities [13,14]. This poses challenges to the health of older people, while also presenting novel societal and healthcare challenges. Hence, understanding the risk factors associated with ADL capabilities, and implementing interventions to manage and mitigate their decline assume paramount importance.

ADL encompasses six core domains: bathing, dressing and undressing, toileting, mobility, continence, and feeding [15]. These aspects epitomize the fundamental needs of all humans and directly impact the quality of life and satisfaction of older people [16]. As individuals age, their ADL independence gradually diminishes [17], leading to the need for increased caregiving assistance and more nuanced care [18]. At present, informal caregivers, predominantly family members, provide daily living support for older people, often without remuneration [19]. A significant proportion of these caregivers are women [20]. However, given socioeconomic progress and declining birth rates, family-based caregiving roles are waning, thus challenging traditional caregiving paradigms. A comprehensive understanding of the risk factors of ADL for older people can provide crucial insights for policymakers and healthcare providers to enhance functional independence and quality of life in later years.

Researchers have made important contributions to the field of risk assessment of ADL for older people. However, a scarcity of systematic reviews and studies in this domain hinders the effective assessment of advances and insights. As such, a more systematic and thorough analysis of the development, hotspots, and trend directions is necessary to understand emerging trends in this area.

There are advantages to different review methodologies. However, while traditional review methods provide researchers with targeted and flexible analyses of a small number of published materials [21], bibliometric analysis is a quantitative method used to aggregate an exhaustive bibliography that is widely utilized to identify research trends, expert collaborations, and innovative methodologies [22]. Bibliometrics exceeds the constraints of artificial subjective factors that limit traditional review methods [20,23], enabling a broader, more systematic perspective on the scope and trajectory of research within a given field [24,25].

To our knowledge, this study is the first to comprehensively explore hotspots, trends, and future directions in the risk assessment of ADL in older people. The present work aims to fill a gap in the bibliometric reviews of this topic by (i) summarizing research on risk assessment of ADL in older people from 2004 to 2013, (ii) elucidating prevalent research themes and their attributes in the field, and (iii) analyzing research avenues based on emerging trends.

## 2. Material and Methods

### 2.1. Data Retrieval and Processing

Web of Science (WoS), managed by Clarivate Analytics in the USA [26,27], is the world’s most authoritative citation database, renowned for its stringent standards and extensive multidisciplinary coverage [28]. It supports major international languages, including English, French, German, Spanish, Mandarin, Arabic, and other major world languages, and includes significant databases such as the Science Citation Index Expanded (SCIE), the Social Sciences Citation Index (SSCI), the Arts & Humanities Citation Index (AHCI), and the Emerging Sources Citation Index (ESCI) [29,30]. Each database is tailored to compile the literature from the respective fields of science, social science, arts and humanities, and newly emerging areas, respectively. Journals included in these databases must adhere to rigorous standards, including a thorough peer review process and an emphasis on citation impact [28]. Moreover, a notable feature of the WoS core collection is its citation counts, which offer an objective metric for assessing the relative importance of scholarly articles [28,31]. Currently, WoS is widely used for bibliometric analysis [31,32,33,34,35,36,37]. Furthermore, this study utilizes the bibliometric tool CiteSpace, which was originally designed for use with the WoS database, to effectively leverage its citation data for scientific research and analysis [38,39]. As such, the use of WoS as the data source for this study is fully reasonable.

**Figure 1:**
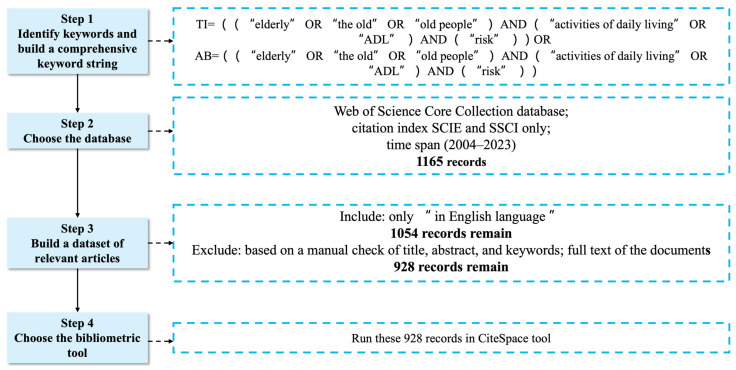
The process of this research.

The literature screening process and inclusion criteria are demonstrated in Figure 1. Two databases from the Web of Science Core Collection were utilized as our primary data sources—the Science Citation Index Expanded (SCIE) and the Social Sciences Citation Index (SSCI). Other databases, such as the Arts & Humanities Citation Index, were not considered as they did not align with the scientific focus of our investigation. The following Boolean search query was employed on 30 March 2024: [TI = ((“elderly” OR “the old” OR “old people”) AND (“activities of daily living” OR “ADL”) AND (“risk”)) OR AB = ((“elderly” OR “the old” OR “old people”) AND (“activities of daily living” OR “ADL”) AND (“risk”))]. This search query yielded 1165 documents (1093 articles, 59 reviews, 6 conference abstracts, and 7 other document types), which were then filtered based on language; only those written in English were retained, resulting in 1054 documents. After thorough examination of article titles, abstracts, and contents, irrelevant articles were excluded, leaving a final selection of 928 articles for bibliometric and visual analysis.

The articles were initially obtained in plain text format, encompassing complete records and references. These were imported into CiteSpace 6.1.R6 for further analysis. CiteSpace is a literature-based analysis method consisting of a number of procedural steps, including time slicing, threshold setting, modeling, pruning, merging, and mapping, to visualize digital information. Core concepts within the program such as burst detection, centrality, and heterogeneous networks, facilitate timely visualization of research status, hotspots, and frontiers [40]. Furthermore, CiteSpace elucidates connections or working relationships between documents, assisting users in bridging cognitive gaps and identifying key points and future research trends within domains [41].

The CiteSpace method was employed to analyze existing research pertaining to the risk assessment of ADL for older people. Additionally, an in-depth reading was conducted to thoroughly analyze current research hotspots, frontiers, and trends, and provide insights into current and future research directions.

### 2.2. Bibliometric and Visual Analysis Methods

The retrieved literature was organized and stored using the analysis retrieval function of Web of Science https://www.webofscience.com/wos/woscc/basic-search (30 March 2024). Statistical analysis on annual publication volume, publication countries, publishing institutions, and authors was conducted using Microsoft Excel 16.63.1 (2022). Furthermore, CiteSpace (version 6.1.R6) was employed to extract target noun phrases from titles, abstracts, and keywords for co-word analysis, co-occurrence analysis, and burst word analysis. Both programs were utilized for plotting to generate a development trajectory and research hotspot map for the risk assessment of ADL for older people. We chose a 20-year time span in CiteSpace, with time nodes set at 1-year intervals. Node types included Country and Keyword, with node strength defaulted to cosine. The threshold was set to TOP 20, and the network pruning function was configured to Minimum Spanning Tree for graph analysis.

## 3. Results

### 3.1. Literature Quantity Analysis

**Figure 2:**
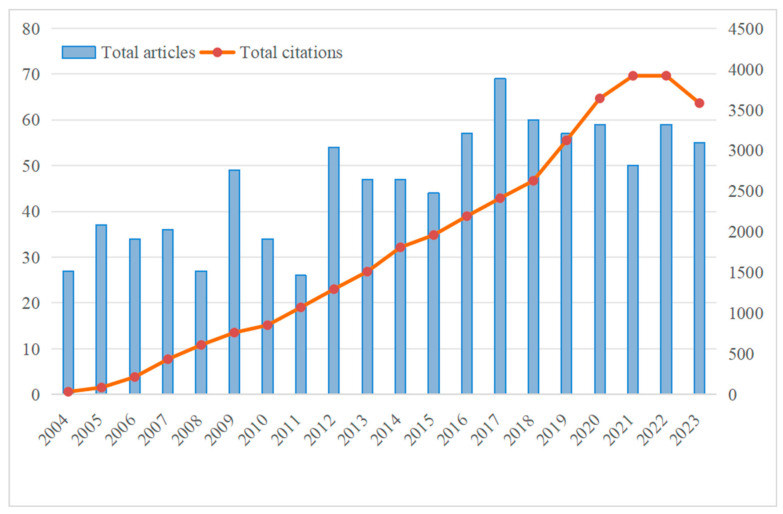
Temporal evolution of total publications.

Figure 2 illustrates the yearly distribution of literature on the topic of risk assessment of ADL for older people within the Web of Science Core Collection from 2004 to 2023. Results show that, on average, only 46 articles were published on this subject annually. In recent years, there has been a modest increase in both publication count and citations. The increase in publications has been gradual, rising from an initial 27 articles to 55 articles. Meanwhile, citations exhibit a sustained upward trajectory, despite a slight decline in 2023, with an overall increase from 34 in 2004 to 3581 in 2022.

While the publication frequency of research remains relatively low, the field is experiencing rapid development. However, according to the results shown in Figure 2, there remains substantial room for further exploration in the coming years, indicating a continued growth trajectory.

### 3.2. National (Regions), Institutional, and Disciplinary Analysis

#### 3.2.1. Visualization of National Research (Regions)

**Figure 3:**
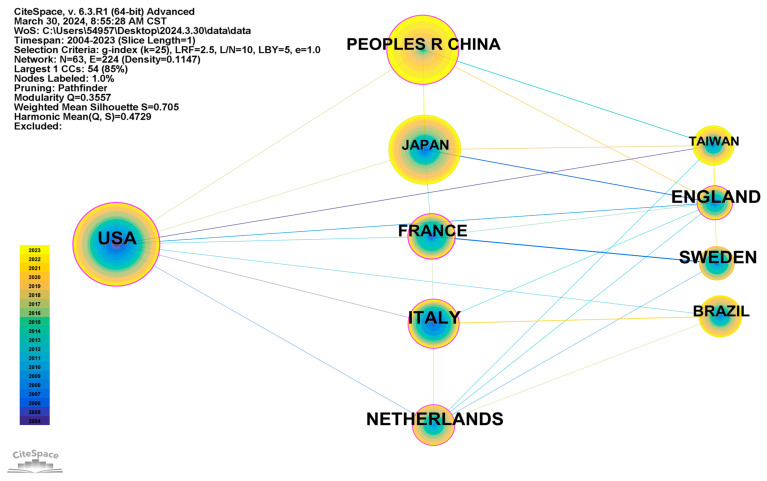
Collaborative network diagram of the top ten countries (regions) in the field of activities of daily living risk among older people.

A quantitative analysis identified core publishing countries in this research field, while reflecting academic exchanges and collaborations between countries. CiteSpace was employed to analyze countries as research objects, with time slicing spanning 2004 to 2023, setting one year per slice, and applying a threshold of the top 20. This resulted in a network map of 63 nodes and 224 links, with a density of 0.1147 (Figure 3). The thickness of the purple circles on the map corresponds to the degree of betweenness centrality, while the centrality of nodes gauges the significance of their positions within the network.

Table 1 presents the 10 countries with the highest publication frequency. Frequency denotes the number of occurrences of each country, while centrality signifies the position of the country within the field. Evidently, centrality mirrors the connections between countries. Specifically, countries with a greater number of connections exhibit higher centrality, signifying more substantial research output and a more prominent position in the field.

The United States has a publication frequency of 186, more than all other nations (Table 1). With a centrality of 0.29, the United States exhibits relatively widespread international collaborations. Japan had the second highest publication frequency of 142, while the People’s Republic of China had the third highest publication frequency of 137. However, despite their high publication frequencies, the latter two countries demonstrate marked centrality differences from the United States, indicating relatively fewer international collaborations. Japan, Brazil, and Taiwan exhibit the lowest centrality, suggesting less international collaboration when researching the topic. Conversely, the centrality of the People’s Republic of China, Italy, France, the Netherlands, and England exceeds 0.1, highlighting the collaboration between multinational researchers. The United States, Japan, and the People’s Republic of China all published articles in this field as far back as 2004, and their sustained citation frequencies underscore their foundational roles in this field.

#### 3.2.2. Visualization of Disciplinary Collaboration

**Figure 4:**
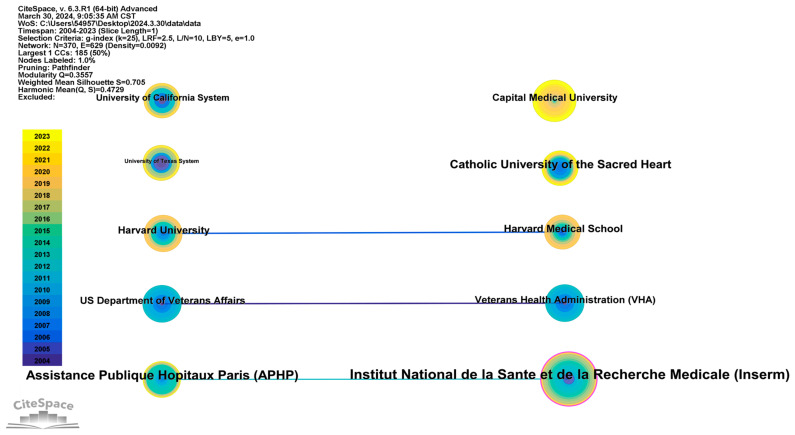
Collaboration network of the top ten institutions in the field of activities of daily living risk among older people.

A CiteSpace analysis of institutions generated a network map comprising 370 nodes and 629 links, with a density of 0.0092 (Figure 4). As illustrated in Figure 4, the network nodes are relatively densely interconnected, with a total of 629 links, indicating extensive collaboration between most of the included research institutions and notable regional characteristics. Notably, only Institut National de la Sante et de la Recherche Medicale (Inserm) in France exhibits a centrality exceeding 0.1, suggesting relatively common inter-institutional collaborations. This underscores the need to promote sustained cross-institutional research and collaboration to foster academic exchange within this field.

Furthermore, we compiled a list of the 10 institutions with the most publications (Table 2). Institut National de la Sante et de la Recherche Medicale (Inserm), with 34 publications, emerged as the most prolific institution. An examination of publication years reveals that the contributions of this institution to research on the risk assessment of ADL for older people date back to 2004, highlighting its significant role in advancing this field. Capital Medical University in China has the second highest number of publications, with a frequency of 21, and publications dating back to 2004. Although Capital Medical University paid early attention to this research field and currently ranks second in publication frequency, its centrality is only 0.04, indicating relatively fewer cross-institutional collaborations in recent years. Notably, although the Catholic University of the Sacred Heart in Italy has only 15 publications, its centrality is relatively high at 0.09. In other words, this institution has conducted in-depth research in this field recently, pointing to directions of current frontier research.

#### 3.2.3. Visualization Analysis of Disciplines

**Figure 5:**
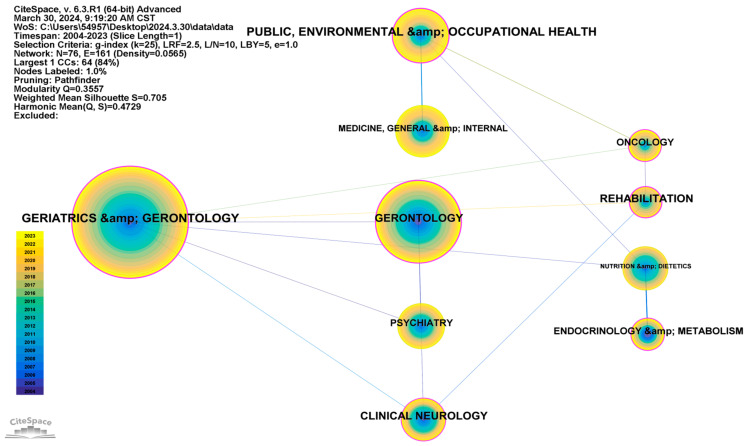
Collaboration network of the top ten disciplines in the field of activities of daily living risk among older people.

A frequency analysis, as shown in Figure 5 and Table 3, reveals that Geriatrics and Gerontology is the discipline with the most publications in this field of research, with a frequency of 353. This indicates the focus of this discipline on research into the risk assessment of ADL for older people. Geriatrics and Gerontology is the primary and representative discipline in this field of study and has a centrality of 0.32.

The field of Gerontology has the second highest publication frequency with 186 publications, while Public, Environmental, and Occupational Health has the third highest with 92 publications. This latter discipline, despite ranking third in publication frequency, has a centrality of 0.38, similar to Geriatrics and Gerontology. This suggests relatively common interdisciplinary cooperation between these two disciplines, establishing the groundwork for research in this field.

### 3.3. Analysis of Current Research Hotspots and Frontier Trends

#### 3.3.1. Current Major Research Hotspots

**Figure 6:**
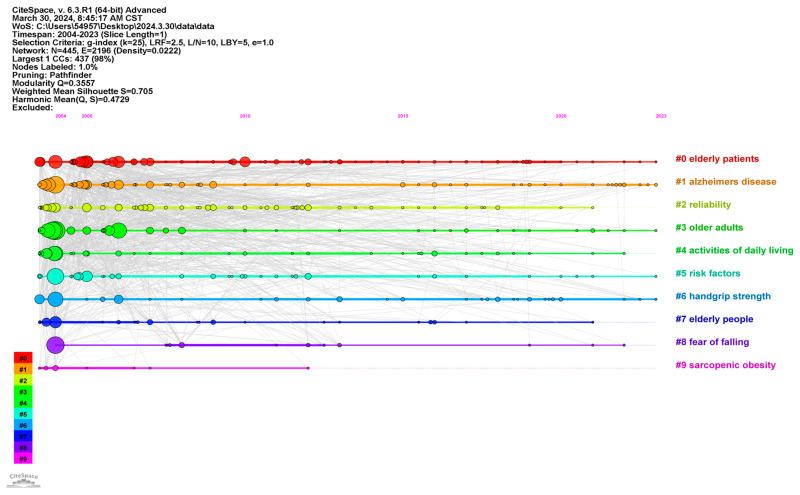
Analysis of research hotspots in the field of activities of daily living risk among older people.

We used CiteSpace to perform keyword cluster analysis, utilizing the cluster option and implementing the Pathfinder algorithm to refine linkages, ensuring the coherence of clustering. Figure 6 illustrates the most common research themes in the domain of risk assessment of ADL for older people. The cluster numbers correspond to the themes derived from keyword clustering using the LLR algorithm, resulting in a total of 10 clusters. Detailed information for each cluster is depicted in Figure 6.

In Figure 6, each node represents a keyword. A total of 445 keywords were obtained (N = 445), with an association frequency of 2196 (E = 2196) between keywords. This generated 10 research themes: #0 elderly patients; #1 Alzheimer’s disease; #2 reliability; #3 older adults; #4 activities of daily living; #5 risk factors; #6 handgrip strength; #7 elderly people; #8 fear of falling; and #9 sarcopenic obesity. In Figure 6, different clusters represent different keywords, and the year indicates the time of publication for keywords within each cluster, which also indicates whether the theme is close to the research frontier in this field. Notably, “elderly patients”, “Alzheimer’s disease”, and “older adults” are the most common research topics (Figure 6).

We identified research hotspots by integrating the co-occurring keywords from these 10 clusters. Two hotspots emerged: (1) risk factors and effective interventions for the decline in older people’s ADL abilities, and (2) assessment of diseases related to ADL abilities in older people.

Clusters #2, #3, #4, #5, and #7 specifically focus on the risk of decline in older people’s ADL and related intervention measures. During the aging process, physiological factors (gender [42], age [43,44,45], cognitive impairment [46], nutritional intake [47,48], chronic diseases [49,50]), psychological factors (loneliness [51] depression [9,52]), and social factors (income [53], marital status [44,54], social support [55,56]) contribute to the ability to engage in ADL. Physical exercise training interventions [57,58,59], cognitive interventions [2,60], and community interventions [61] effectively support various ADL abilities in older people.

Clusters #0, #1, #6, #8, and #9 focus on the assessment, diagnosis, and treatment of diseases related to ADL among older people. The following diseases are particularly prominent among older people with limited abilities to engage in ADL: (1) Neurocognitive disorders: Research on cognitive impairment, dementia, Alzheimer’s disease, and depression has been ongoing for decades, with the prevalence of such neurocognitive disorders significantly increasing in the older population [62]; (2) Studies related to impaired muscle function have become a specific research area. Impaired muscle function, functional limitations in ADL, and disability often coexist [63,64,65,66,67]. Loss of muscle mass, muscle weakness, sarcopenia, and sarcopenic obesity are common geriatric syndromes [68,69], which often lead to bone instability [70,71,72] and increase the risk of falls and fractures [73]; and (3) Falls and fractures: Research on falls and fractures is also an emerging field. Fall studies include accidental falls and fear of falling, while fracture studies particularly focus on hip fractures and femoral fractures. The relevant literature focuses on treatment procedures, prognosis, and preventive measures.

#### 3.3.2. Future Research Frontiers

We utilized the CiteSpace burst detection algorithm to analyze changes in research topics and identify hotspots. This generated an evolution map of keyword hotspots from the Web of Science database with specific parameters and threshold settings, including a time slice set to 1, and a threshold selection of g-index = 5. Additionally, smaller keyword nodes were concealed to produce a simplified map (Table 4). Blue lines represent keyword bursts, while red line segments denote periods of significant emergence, in particular thematic categories, indicating the start and end years of the burst duration.

The timeline illustrates the appearance of keywords over time, with the position of each keyword representing the year of its first appearance. As shown in Table 4, the distribution of keywords related to the risks of ADL for older people is relatively uniform, indicating diverse research themes in different periods, and continuous enrichment of research. These themes can be divided into four stages: (1) From 2004 to 2008, the research hotspots were population, community, Alzheimer’s disease, and functional status. This suggests that the field began with a demographic focus. (2) From 2009 to 2014, the common research topics were prevention, disease, frail elderly, and cardiovascular disease. During this stage, the risk of ADL among older people was studied from a geriatric medicine perspective. (3) From 2015 to 2019, research hotspots included geriatric assessment, frailty, heart failure, and exercise. This indicates a deeper exploration of different domains of ADL, focusing on assessments of older people’s independence in specific areas. (4) The year 2019 was a pivotal point, with the emergence of new topics and hotspots such as sarcopenia, hip fracture, cognitive function, machine learning, and instrumental activity. This suggests that the assessment of risk factors and diseases related to ADL is now at the forefront, with machine learning an increasingly popular research approach.

## 4. Discussion

Researchers have shown a growing interest in studying the risks for older people associated with ADL. However, there remains a notable gap in the literature concerning comprehensive reviews and future outlooks in this field. Traditional review studies are hindered by a reliance on a limited pool of publications, which may introduce bias and limitations due to personal and subjective selection criteria. In contrast, bibliometric analysis comprehensively covers all the relevant literature within a specified timeframe, thus minimizing subjectivity and avoiding the omission of crucial publications. This method allows for a quantitative exploration of the knowledge structure, research hotspots, and emerging insights within a specific scientific field [22,74]. Consequently, this study utilized CiteSpace software and employed bibliometric techniques to analyze 928 articles from the Web of Science Core Collection database. Our analyses encompassed publication volume, international collaboration, institutional affiliations, disciplinary trends, and keyword patterns to distill key findings and identify research frontiers. Such an approach yielded more objective results to inform future research endeavors.

The key findings of this bibliometric analysis of the risks of ADL for older people are as follows: (1) Publication Trends: The volume of publications in this research domain has shown a steadily increasing trend over the past two decades, with a surge in citations in recent years. This indicates a growing concern for the risks for older people associated with ADL, with the potential for an outbreak trend in the coming years. (2) Geographical Prominence: The United States and certain European countries are prominent in both publication volume and research collaboration in this field. France also exerts significant influence and representation, with the Institut National de la Sante et de la Recherche Medicale (Inserm) occupying a more central position than other institutions. Based on the development and growth in collaboration and exchange between countries and regions in the domain of risks associated with ADL for older people in recent years, such international alliances and collaborations are expected to be a major research trend in the future, requiring strengthened cooperation and interdisciplinary research across regions and institutions. (3) Research Hotspots: Analysis of current research hotspots reveals the centrality of “elderly patients”, “Alzheimer’s disease”, and “older adults” in the field of ADL risks for older people. (4) Future Trends: A summary of burst keywords indicates that “sarcopenia”, “hip fracture”, “cognitive function”, “machine learning”, and “instrumental activity” have emerged as the most popular research topics in recent years. The evaluation of diseases associated with ADL abilities among older people is still actively underway. Particularly noteworthy is the increasing attention paid to machine learning as a research method, which has gradually become a key research focus since its 2021 appearance in the field. It is expected that experts in the field of ADL limitations and machine learning will collaborate more closely in the future. Such interdisciplinary cooperation is essential for enhancing existing tools and creating new ones to assess and improve the ability of older people to undertake ADL. The integration of the analytical capabilities of machine learning with profound insights from seasoned professionals will enhance our understanding of the causes of age-related diseases, and facilitate the development of innovative, predictive methodologies.

In addition to analyzing and discussing the results of the bibliometric study, we undertook critical reading to better elucidate the research topic. Based on our analysis of clusters #2, #3, #4, #5, and #7, it is evident that specific factors may increase the risk of decline in ADL among older people while corresponding interventions focus on assisting with ADL [56]. Consequently, caregiving issues related to older people’s ADL are highly prioritized. However, predictive models for older people’s ADL primarily focus on clinical variables, which are difficult to implement for informal caregivers such as family members, who provide the majority of ADL caregiving [75,76]. Future research should therefore segment the field of daily caregiving to focus on informal caregivers, identify their specific caregiving needs with regard to ADL, and target interventions accordingly.

Furthermore, based on the analysis of clusters #0, #1, #6, #8, and #9, areas of disease research within the ADL risk field deserve attention. ADL limitations are closely linked to physical function-related diseases (such as sarcopenia [77], fractures [8,14,78], chronic pain [79], and falls [7,80]) and neurocognitive disorders (such as dementia [81], cognitive impairment [44], and depression [5,9]). Research into the relationship and potential risks between ADL limitations and these diseases is increasing. Among older people, ADLs are more susceptible to physical function impairment and diseases [7], and the occurrence of related diseases or postoperative complications makes their caregiving tasks [82,83], as well as their prognosis, treatment outcomes, and even mortality, risk more complex [5,7,84,85,86]. While there is consensus in the field regarding the relationship between ADL and related diseases, most studies are cross-sectional or observational, lack causal inference for relevant variables, and contain insufficient evidence. The results of keyword burst analysis from 2021 to 2023 indicate that machine learning as a research method has received significant attention in recent years, as noted earlier. It can help model information based on causal and/or statistical data, potentially revealing hidden dependencies between factors and diseases in a big data environment [87]. In other words, machine learning methods can be used to conduct more in-depth research on aspects of diseases related to ADL for older people.

Despite growing research on this topic, some researchers suggest that more attention should be paid to the design of research methods in the conduct of similar studies [79,83]. From the perspective of clusters #0, #3, #7, and co-occurring keyword analysis, we also found men and women to exhibit different manifestations during the process of ADL decline [79,86]. Relevant studies indicate that biologically, males report faster aging rates [88], while females report higher levels of bodily pain [89,90,91]. As aging becomes a more pressing global issue, a detailed understanding of the related risk factors and adverse outcomes of ADL in older men and women is crucial for clinical guidance, and to maximize the reduction of disabilities. More research is therefore needed to ensure the effective design of research methods.

This study contributes additional evidence concerning the risks associated with ADL among older people, with significance for healthcare professionals and researchers. These findings can assist healthcare providers in prioritizing the assessment of ADL and diseases affecting ADL, thereby improving quality of life and promoting healthy aging among older people. The collaboration of more institutions and disciplines is also needed to enhance international and interdisciplinary exchanges and development.

Despite its contributions, our study has certain limitations. Firstly, the study relied solely on the Web of Science Core Collection database, which potentially excluded articles not indexed within it. Nevertheless, this database is widely recognized for its high-quality literature. Secondly, due to the limited available literature in this field, our bibliometric analysis results may not fully capture all aspects of the research domain. Moreover, the definition of older people varies across different countries and regions, with some defining older people as those aged 60 years or older, whereas others use 65 years or older as the threshold. These differences can affect the interpretation of our results, as healthy life expectancy and life expectancy may vary accordingly. Future research should therefore include a more detailed consideration of these definitional differences among countries and regions while distinguishing between them in data analysis and interpretation. This will help to more accurately assess the specific challenges and outcomes related to older people’s daily living activities across different nations. Further research should also employ more comprehensive search strategies to validate findings and provide a broader perspective on the risks associated with ADL for older people.

Nevertheless, to the best of our knowledge, few attempts have been made to assess the hotspots and frontiers of research in this domain using comprehensive bibliometric analysis. Given that aging is a global public health priority, scientific metrics analysis methods can review the developmental trajectory of this field, predict future research hotspots, and provide guidance for researchers conducting related studies.

## 5. Conclusions

This study is a first attempt to conduct a comprehensive bibliometric analysis of more than 20 years of published research regarding risks associated with ADL for older people. The study used the knowledge graph software CiteSpace to elucidate the overall structure and developmental trajectory of research in this domain. Significant progress has been made in the research on risks associated with ADL among older people. Studies in this field have primarily focused on the assessment of diseases related to the ADL of older people, and future hotspots and trends are expected to include the development of preventive mechanisms for assessing trends in their ADL, caregiving needs, and the prevention of adverse outcomes.

Furthermore, a notable limitation in this field of research is the lack of international and cross-regional collaborative efforts. Researchers should strive to leverage modern high-tech tools to strengthen cross-regional and interdisciplinary collaboration in the comprehensive exploration of preventive mechanisms for the risks associated with the activities of daily living for older people.

## Figures and Tables

**Table 1:** Collaboration relationships of the top ten countries (regions) in the field of activities of daily living risk among older people.

Count	Centrality	Year	Country/Region
186	0.29	2004	USA
142	0.02	2004	JAPAN
137	0.1	2004	PEOPLES R CHINA
69	0.11	2004	ITALY
60	0.22	2004	FRANCE
53	0.13	2005	NETHERLANDS
47	0.02	2007	BRAZIL
45	0	2004	TAIWAN
37	0.07	2004	SWEDEN
35	0.19	2004	ENGLAND

USA: the United States of America; PEOPLES R CHINA: the People’s Republic of China.

**Table 2:** Collaboration relationships of the top ten institutions in the field of activities of daily living risk among older people.

Count	Centrality	Year	Institutions
34	0.11	2004	Institut National de la Sante et de la Recherche Medicale (Inserm)
21	0.04	2004	Capital Medical University
18	0.02	2005	Harvard University
18	0.03	2004	Assistance Publique Hopitaux Paris (APHP)
16	0.02	2004	Veterans Health Administration (VHA)
16	0.02	2004	US Department of Veterans Affairs
15	0.08	2005	Catholic University of the Sacred Heart
14	0.05	2005	Harvard Medical School
14	0.01	2004	University of California System
14	0.01	2004	University of Texas System

**Table 3:** Collaboration relationships of the top ten disciplines in the field of activities of daily living risk among older people.

Count	Centrality	Year	Categories
353	0.32	2004	Geriatrics and Gerontology
186	0.22	2004	Gerontology
92	0.38	2004	Public, Environmental, and Occupational Health
83	0.04	2004	Medicine, General and Internal
63	0.07	2004	Psychiatry
60	0.03	2004	Nutrition and Dietetics
48	0.17	2004	Clinical Neurology
31	0.15	2004	Oncology
31	0.16	2004	Endocrinology and Metabolism
30	0.21	2004	Rehabilitation

**Table 4:** Top 20 keywords with the strongest citation bursts.

Top 20 Keywords with the Strongest Citation Bursts				
Keywords	Strength	Begin	End	2004–2023
Population	4.21	2004	2006	▃▃▃ ▂▂▂▂▂▂▂▂▂▂▂▂▂▂▂▂▂
Community	5.31	2005	2008	▂ ▃▃▃▃ ▂▂▂▂▂▂▂▂▂▂▂▂▂▂▂
Alzheimer’s disease	4.32	2005	2007	▂ ▃▃▃ ▂▂▂▂▂▂▂▂▂▂▂▂▂▂▂▂
Functional status	3.49	2005	2006	▂ ▃▃ ▂▂▂▂▂▂▂▂▂▂▂▂▂▂▂▂▂
Mortality	3.79	2007	2008	▂▂▂ ▃▃ ▂▂▂▂▂▂▂▂▂▂▂▂▂▂▂
Illness	4.37	2008	2015	▂▂▂▂ ▃▃▃▃▃▃▃▃ ▂▂▂▂▂▂▂▂
Prevention	5.02	2009	2011	▂▂▂▂▂ ▃▃▃ ▂▂▂▂▂▂▂▂▂▂▂▂
Disability	4.54	2009	2012	▂▂ ▂▂▂ ▃▃▃▃ ▂▂▂▂▂▂▂▂▂▂▂
Frail elderly	3.34	2010	2013	▂▂▂▂▂▂ ▃▃▃▃ ▂▂▂▂▂▂▂▂▂▂
Cardiovascular disease	3.21	2010	2014	▂▂▂▂▂▂ ▃▃▃▃▃ ▂▂▂▂▂▂▂▂▂
Depression	3.53	2014	2016	▂▂▂▂▂▂▂▂▂▂ ▃▃▃ ▂▂▂▂▂▂▂
Geriatric assessment	4.51	2015	2019	▂▂▂▂▂▂▂▂ ▂▂▂ ▃▃▃▃▃ ▂▂▂▂
Frailty	4.49	2016	2019	▂▂▂▂▂▂ ▂▂▂▂▂▂ ▃▃▃▃ ▂▂▂▂
Heart failure	3.94	2016	2019	▂▂▂▂▂▂▂▂▂▂▂▂ ▃▃▃▃ ▂▂▂▂
Exercise	5.32	2018	2019	▂▂▂▂▂ ▂▂▂▂▂▂▂▂▂ ▃▃ ▂▂▂▂
Sarcopenia	3.69	2018	2023	▂▂▂▂▂▂▂▂▂ ▂▂▂▂▂ ▃▃▃▃▃▃
Hip fracture	3.52	2019	2023	▂ ▂▂▂▂▂▂▂▂▂▂▂▂▂▂ ▃▃▃▃▃
Cognitive function	3.12	2019	2023	▂▂▂▂▂▂▂▂▂▂▂▂ ▂▂▂ ▃▃▃▃▃
Machine learning	3.54	2021	2023	▂▂▂▂▂▂▂▂▂▂▂▂▂▂▂▂▂ ▃▃▃
Instrumental activity	3.19	2021	2023	▂ ▂▂▂▂▂▂▂▂▂▂▂▂▂▂▂▂ ▃▃▃

## Data Availability

The data that support the findings of this study are available from the first author upon reasonable request.
